# Myocarditis or Pericarditis Events After BNT162b2 Vaccination in Individuals Aged 12 to 17 Years in Ontario, Canada

**DOI:** 10.1001/jamapediatrics.2022.6166

**Published:** 2023-02-27

**Authors:** Sarah A. Buchan, Sarah Alley, Chi Yon Seo, Caitlin Johnson, Jeffrey C. Kwong, Sharifa Nasreen, Nisha Thampi, Diane Lu, Tara M. Harris, Andrew Calzavara, Sarah E. Wilson

**Affiliations:** 1 Public Health Ontario, Toronto, Ontario, Canada; 2 Dalla Lana School of Public Health, University of Toronto, Toronto, Ontario, Canada; 3 ICES, Toronto, Ontario, Canada; 4 Department of Family and Community Medicine, University of Toronto, Toronto, Ontario, Canada; 5 University Health Network, Toronto, Ontario, Canada; 6 Department of Pediatrics, University of Ottawa, Ottawa, Ontario, Canada

## Abstract

**Importance:**

The risk of myocarditis or pericarditis after COVID-19 messenger RNA vaccines varies by age and sex, and there is some evidence to suggest increasing risk with shorter intervals between dose 1 and 2 (ie, interdose interval).

**Objective:**

To estimate the incidence of reported myocarditis or pericarditis after BNT162b2 vaccine among adolescents and to describe the clinical information associated with these events.

**Design, Setting, and Participants:**

This was a population-based cohort study using passive vaccine safety surveillance data linked to the provincial COVID-19 vaccine registry. Included in the study were all adolescents aged 12 to 17 years in Ontario, Canada, who received 1 or more doses of BNT162b2 vaccine between December 14, 2020, and November 21, 2021, and reported an episode of myocarditis or pericarditis. Data were analyzed from December 15, 2021, to April 22, 2022.

**Exposure:**

Receipt of BNT162b2 (Comirnaty [Pfizer-BioNTech]) vaccine.

**Main Outcomes and Measure:**

Reported incidence of myocarditis or pericarditis meeting level 1 to 3 of the Brighton Collaboration case definition per 100 000 doses of BNT162b2 administered by age group (12-15 years vs 16-17 years), sex, dose number, and interdose interval. All clinical information associated with symptoms, health care usage, diagnostic test results, and treatment at the time of the acute event were summarized.

**Results:**

There were approximately 1.65 million doses of BNT162b2 administered and 77 reports of myocarditis or pericarditis among those aged 12 to 17 years, which met the inclusion criteria during the study period. Of the 77 adolescents (mean [SD] age, 15.0 [1.7] years; 63 male individuals [81.8%]), 51 (66.2%) developed myocarditis or pericarditis after dose 2 of BNT162b2. Overall, 74 individuals (96.1%) with an event were assessed in the emergency department, and 34 (44.2%) were hospitalized (median [IQR] length of stay, 1 [1-2] day). The majority of adolescents (57 [74.0%]) were treated with nonsteroidal anti-inflammatory drugs only, and 11 (14.3%) required no treatment. The highest reported incidence was observed among male adolescents aged 16 to 17 years after dose 2 (15.7 per 100 000; 95% CI, 9.7-23.9). Among those aged 16 to 17 years, the reporting rate was highest in those with a short (ie, ≤30 days) interdose interval (21.3 per 100 000; 95% CI, 11.0-37.2).

**Conclusions and Relevance:**

Results of this cohort study suggest that there was variation in the reported incidence of myocarditis or pericarditis after BNT162b2 vaccine among adolescent age groups. However, the risk of these events after vaccination remains very rare and should be considered in relation to the benefits of COVID-19 vaccination.

## Key Points

**Question:**

Does the incidence of reported myocarditis or pericarditis after BNT162b2 vaccination vary by age, sex, and interdose interval among adolescents aged 12 to 17 years?

**Findings:**

In this population-based cohort study of 1.65 million doses of BNT162b2 vaccinations among adolescents in Ontario, Canada, there were 77 adolescents with myocarditis or pericarditis after vaccination and a higher reported incidence among adolescents aged 16 to 17 years vs 12 to 15 years and among those aged 16 to 17 years with a shorter interval between doses. Overall, many of the cases of myocarditis and pericarditis were mild, and the adolescents required either no treatment or were treated conservatively with nonsteroidal anti-inflammatory drugs.

**Meaning:**

Study results suggest that there is variation in the reported incidence of myocarditis or pericarditis after BNT162b2 vaccination among adolescent age groups, but the risk remains rare.

## Teaser

This cohort study investigates the association of myocarditis or pericarditis with BNT162b2 vaccination in adolescents aged 12 to 17 years.

## Introduction

Cases of myocarditis and pericarditis after COVID-19 messenger RNA (mRNA) vaccines have been reported internationally as jurisdictions implemented their vaccination programs. Although clinical trials were not adequately powered to detect these rare outcomes, real-world data have demonstrated that the risk is highest in young male individuals, particularly after dose 2.^1,2,3,4,5^ Importantly, there are also known risks of myocarditis after SARS-CoV-2 infection,^6,7,8,9,10^ with studies demonstrating a higher rate of events after infection relative to vaccination overall across ages.^6^

Although an age effect of these events after COVID-19 mRNA vaccination has been consistently observed, more recent data have indicated additional variability within typically used age groupings. Specifically, data from the US and Israel have indicated that younger adolescents (ie, 12-15 years) have lower rates of myocarditis or pericarditis after vaccination than older adolescents (ie, 16-17 years or 16-19 years).^1,11,12,13^ Fortunately, episodes of myocarditis or pericarditis in most adolescents who have received COVID-19 mRNA vaccines appear to resolve quickly with ambulatory treatment with nonsteroidal anti-inflammatory drugs (NSAIDs),^14,15,16^ with some data to suggest that events occurring after vaccination are less severe than episodes of myocarditis or pericarditis after SARS-CoV-2 infection.^17,18,19^

In Ontario, Canada, BNT162b2 (Comirnaty [Pfizer-BioNTech]) has been exclusively used as the product for the primary series in those aged 12 to 17 years; 2-dose coverage for this age group is high, with approximately 75% having completed their primary series by November 2021.^20^ When the broad adolescent vaccine program started in May 2021, an extended interval (ie, 8 weeks) between dose 1 and dose 2 was in use due to vaccine supply constraints. As supply increased, this interval was reduced over time, and by late summer 2021, a 4-week interval was used.^21,22^ A return to the recommended 8-week interval occurred again after recommendations from Canada’s National Advisory Committee on Immunization that were issued in October 2021.

The objectives of this study were to (1) examine the characteristics, presenting symptoms, clinical investigations (ie, laboratory and diagnostic imaging); (2) report incidence of myocarditis or pericarditis after BNT162b2 vaccination in individuals aged 12 to 17 years; and (3) compare reported incidence between those aged 12 to15 years and 16 to 17 years overall and by interdose interval, in order to better understand the epidemiology of these events in adolescents and to inform vaccine program decisions.

## Methods

The Public Health Ontario ethics review board determined that this study did not require research ethics committee approval as the activities described in this article were conducted in fulfillment of Public Health Ontario’s legislated mandate “…to provide scientific and technical advice and support to the health care system and the Government of Ontario in order to protect and promote the health of Ontarians…”^23^ and are therefore considered public health practice, not research. Data were deidentified, and informed consent was, therefore, not required. This study followed the Strengthening the Reporting of Observational Studies in Epidemiology (STROBE) reporting guidelines.^24^

Reporting of adverse events following immunization (AEFI) in Ontario is mandated by provincial public health legislation and is primarily done by health care professionals, although voluntary reporting from vaccine recipients or their caregivers also occurs. Local public health units (PHUs) investigate and report AEFIs using provincial surveillance criteria. The PHU investigation involves requesting and reviewing medical records, including laboratory and diagnostic findings, if available, and entering this information into the centralized database used for COVID-19 passive vaccine safety surveillance. A standardized data collection tool to assist PHUs in the collection of relevant information from the medical chart was developed to support enhanced surveillance of myocarditis and pericarditis after mRNA COVID-19 vaccines in Ontario.

We used Ontario’s passive vaccine safety surveillance data to estimate rates of myocarditis or pericarditis after vaccination with BNT162b2, as described previously.^25^ Briefly, we extracted data from the provincial reporting system on all myocarditis or pericarditis adverse events after COVID-19 vaccination that were reported for adolescents aged 12 to 17 years in Ontario, who received 1 or more doses of BNT162b2 vaccine between December 14, 2020 (the start of the overall vaccine program in Ontario), and November 21, 2021. Events were identified using a data element to capture myocarditis or pericarditis reports that were added to the provincial system in 2021. Myocarditis or pericarditis events reported before the creation of this drop-down value were migrated to this new response option as part of its implementation. All reports of myocarditis and pericarditis in the provincial database are reviewed by vaccine safety nurses and physicians at Public Health Ontario to assign a level of diagnostic certainty using Brighton Collaboration (BC) case definitions, with level 1 representing the highest level of diagnostic certainty (ie, a definitive case).^26^ We restricted our main analyses to events of myocarditis or pericarditis meeting BC levels 1 to 3. Additionally, we extracted reported events of chest pain without a physician diagnosis of myocarditis or pericarditis in order to quantify these events separately. A key word search was used to identify these reports as there is no drop-down data element in the provincial surveillance system to capture this specific event. Clinical information documented in the provincial AEFI system according to the AEFI investigation was summarized for all events of myocarditis and pericarditis meeting BC levels 1 through 3 and included presenting symptoms, health care usage, diagnostic test results, and treatment information available at the time of the acute event. Ontario’s COVID-19 public health case and contact management database, a centralized database documenting all laboratory-confirmed COVID-19 cases, was used to determine past history of SARS-CoV-2 infection, based on positive reverse transcription–polymerase chain reaction (PCR) test results and associated date. We extracted denominator data from COVaxON, the provincial COVID-19 vaccine registry, to calculate rates and to verify dose number, product, and dates of vaccination for interval calculations. Participant race and ethnicity data are not routinely collected as part of AEFI investigations in Ontario and therefore were not available to report.

### Statistical Analysis

We calculated the reported incidence per 100 000 doses of BNT162b2 administered by age group (12-15 years vs 16-17 years) and sex, as well as by dose number and the interval (number of days) between dose 1 and 2 (interdose interval hereafter). We calculated 95% CIs using the exact Poisson method, and significance was assessed using nonoverlapping CIs. We repeated the overall incidence calculations by dose number excluding individuals with a prior history of PCR-confirmed SARS-CoV-2 infection. We also performed a sensitivity analysis repeating the incidence calculations restricted to events that occurred within 7 days of vaccine administration.

Finally, we compared observed vs expected cases of myocarditis or pericarditis (using a 7-day risk interval) based on historical data on emergency department visits and hospitalizations during the 2015 to 2019 period obtained from ICES (formerly Institute for Clinical Evaluative Sciences), as previously described.^27^ These data reflect all reports of myocarditis and pericarditis in these settings for the province, where health care is provided universally under a single payer system. We calculated a risk difference (with 95% CI) per 100 000 doses by subtracting the expected cases from observed cases, for each strata of age, sex, and dose number. We repeated this analysis excluding 2 adolescents who were not seen in the emergency department nor hospitalized, in order to make the observed events more comparable with the historical background data. All tests were 2-sided and used a significance level of *P* < .05. Data were analyzed using SAS Enterprise Guide, version 8.2 (SAS Institute) from December 15, 2021, to April 22, 2022.

## Results

Between December 14, 2020, and November 21, 2021, there were approximately 1.65 million doses of BNT162b2 administered to those aged 12 to 17 years in Ontario, and 205 episodes of chest pain with or without a diagnosis of myocarditis or pericarditis after vaccination were reported in those aged 12 to 17 years, representing 36.1% of AEFI reports in this age group. Of these, there were 100 AEFI reports of chest pain for whom there was no physician diagnosis of myocarditis or pericarditis, either because cardiac investigations were all normal or were not ordered, 28 AEFI reports of myocarditis of pericarditis classified as BC level 4, and 77 reported events that met BC level 1 to 3.

Of the 77 adolescents who met inclusion criteria (mean [SD] age, 15.0 [1.7] years; 63 male individuals [81.8%]; 14 female individuals [18.2%]), 26 (33.8%) developed myocarditis or pericarditis after dose 1 of BNT162b2, and 51 (66.2%) developed myocarditis or pericarditis after dose 2 of BNT162b2 (Table 1). Most events occurred within 7 days of vaccine administration (16 [64.0%] for dose 1 and 49 [96.1%] for dose 2). The median (IQR) time to symptom onset after dose 2 was 2 (2-3) days. Most adolescents (39 [50.6%]) were diagnosed with myocarditis, followed by myopericarditis/perimyocarditis (22 [28.6%]) and pericarditis (16 [20.8%]). All 22 adolescents who were diagnosed with myopericarditis/perimyocarditis met BC criteria level 1 to 3 for myocarditis.

**Table 1.** Characteristics of Adolescents Meeting Brighton Collaboration Levels 1 to 3 for Myocarditis or Pericarditis After Vaccination With BNT162b2

Characteristics	Year interval, No. (%)		Overall
	12-15	16-17	
No.	39	38	77
Sex			
Male	31 (79.5)	32 (84.2)	63 (81.8)
Female	8 (20.5)	6 (15.8)	14 (18.2)
Dose No.			
Dose 1	11 (28.2)	15 (39.5)	26 (33.8)
Time to onset, median (IQR), d	5 (2-20)	3 (2-14) 1	4 (2-14)
Events within 7 d after dose 1	7 (63.6)	9 (64.3)1	16 (64.0)^a^
Dose 2	28 (71.8)	23 (60.5)	51 (66.2)
Time to onset, median (IQR), d	2 (2-3)	2 (1-2)	2 (2-3)
Events within 7 d after dose 2	26 (92.9)	23 (100.0)	49 (96.1)
Clinical diagnosis			
Myocarditis	25 (64.1)	14 (36.8)	39 (50.6)
Pericarditis	5 (12.8)	11 (28.9)	16 (20.8)
Myopericarditis/perimyocarditis	9 (23.1)	13 (34.2)	22 (28.6)

^a^ Excludes 1 event following dose 1 with a missing onset date.

On clinical presentation, the majority of adolescents (74 [96.1%]) presented with chest pain, followed by shortness of breath (33 [42.9%]), gastrointestinal symptoms (eg, nausea, vomiting; 23 [29.9%]), fever (14 [18.2%]), and palpitations (13 [16.9%]) (Table 2). These presenting symptoms were similar across diagnoses of myocarditis, pericarditis, or myopericarditis/perimyocarditis. Overall, 74 adolescents (96.1%) were assessed in the emergency department, and 34 adolescents (44.2%) were hospitalized; among hospitalized individuals, the median (IQR) length of stay was 1 (1-2) day (Table 2). The proportion of adolescents hospitalized ranged from 2 (12.5%) for pericarditis to 21 (53.8%) for myocarditis. Two individuals (2.6%) were admitted to the intensive care unit (ICU), and there were no deaths. Of the 2 adolescents admitted to the ICU, 1 required pericardiocentesis and placement of a pericardial drain, and the other received additional monitoring associated with the extent of troponin release observed; neither of these individuals required inotropic, mechanical, or circulatory support in the ICU.^15^

**Table 2.** Clinical Information for Myocarditis or Pericarditis Events Among Adolescents Aged 12 to 17 Years

Variable	No. (%)			
	Myocarditis	Pericarditis	Myopericarditis/perimyocarditis	Total
No.	39	16	22	77
Symptoms on presentation^a^				
Chest pain	38 (97.4)	15 (93.8)	21 (95.5)	74 (96.1)
Shortness of breath	17 (43.6)	9 (56.3)	7 (31.8)	33 (42.9)
Gastrointestinal	13 (33.3)	4 (25.0)	6 (27.3)	23 (29.9)
Fever	4 (10.3)	4 (25.0)	6 (27.3)	14 (18.2)
Palpitations	7 (17.9)	3 (18.8)	3 (13.6)	13 (16.9)
Dizziness or lightheadedness	6 (15.4)	3 (18.8)	1 (4.5)	10 (13.0)
Diaphoresis	6 (15.4)	0 (0)	0 (0)	6 (7.8)
Fatigue	2 (5.1)	1 (6.3)	2 (9.1)	5 (6.5)
Shoulder, upper back pain	0 (0)	1 (6.3)	2 (9.1)	3 (3.9)
Brighton Collaboration level				
1	1 (2.6)	1 (6.3)	4 (18.2)	6 (7.8)
2	37 (94.9)	14 (87.5)	18 (81.8)	69 (89.6)
3	1 (2.6)	1 (6.3)	0 (0)	2 (2.6)
Health care utilization				
Emergency department	37 (94.9)	15 (93.8)	22 (100)	74 (96.1)
Inpatient hospital admission	21 (53.8)	2 (12.5)	11 (50.0)	34 (44.2)
Length of stay in hospital, median (IQR), d^b^	1 (1-2)	4.5 (3-6)	1.5 (1-2)	1 (1-2)
ICU admission^c^	1 (2.6)	1 (6.3)	0 (0)	2 (2.6)
Death	0 (0)	0 (0)	0 (0)	0 (0)
Diagnostic test results^d^^,^^e^				
Elevated troponin level	36/38 (94.7)	3/15 (20.0)^f^	21/22 (95.5)	60/75 (80.0)
Elevated inflammation biomarker (CRP, ESR, or D-dimer)	17/31 (54.8)	7/14 (50.0)	15/17 (88.2)	39/62 (62.9)
Abnormal echocardiogram	4/34 (11.8)	2/9 (22.2)	6/19 (31.6)	12/62 (19.4)
Reduced LVEF <50%	0/34 (0.0)	0 /9 (0.0)	2/19 (10.5)	2/62 (3.2)
Pericardial effusion	1/34 (2.9)	3/9 (33.3)^g^	4/19 (21.1)	8/62 (12.9)^g^
ECG abnormalities	19/37 (51.4)	14/16 (87.5)	16/22 (72.7)	49/75 (65.3)
Clinical history				
History of myocarditis or pericarditis^h^	0 (0)	0 (0)	0 (0)	0 (0)
Previous COVID-19 RT-PCR positive test result	1 (2.6)	3 (18.8)	1 (4.5)	5 (6.5)^i^
Time between RT-PCR confirmed COVID-19 and vaccination, median, (IQR), s^i^	86 (NA)	162 (40-295)	259 (NA)	162 (86-259)
Treatment				
Only NSAIDs	30 (76.9)	13 (81.3)	14 (63.6)	57 (74.0)
NSAIDs with colchicine^j^	1 (2.6)	2 (12.5)	5 (22.7)	8 (11.7)
No treatment	7 (17.9)	1 (6.3)	3 (13.6)	11 (14.3)
Missing	1 (2.6)	0 (0)	0 (0)	1 (1.3)

Abbreviations: CRP, C-reactive protein; ECG, electrocardiogram; ESR, erythrocyte sedimentation rate; ICU, intensive care unit; LVEF, left ventricular ejection fraction; NSAID, nonsteroidal anti-inflammatory drug; RT-PCR, reverse transcription–polymerase chain reaction.

^a^ Presenting symptoms are not mutually exclusive.

^b^ Restricted to those with known hospitalization dates (excludes 5 hospitalizations with missing dates).

^c^ Of the 2 individuals requiring ICU admission, 1 required pericardiocentesis and placement of a pericardial drain, and the other required further management and monitoring associated with an increase in cardiac enzymes.

^d^ The percentages for these outcomes are restricted to those with nonmissing information. Denominators represent individuals who underwent diagnostic testing and for whom the results were available.

^e^ Two individuals also underwent cardiac magnetic resonance imaging, with findings suggestive of myopericarditis.

^f^ These individuals had elevated troponin levels but were diagnosed as pericarditis by a physician.

^g^ Includes 1 case with pericardial effusion that was identified via a computed tomography scan.

^h^ One individual had unknown or missing history of myocarditis or pericarditis.

^i^ The 5 reported events with a previous positive RT-PCR result were reported after dose 1; of the events reported after dose 2, none had a previous documented RT-PCR result.

^j^ One individual in this group also received an angiotensin-converting enzyme inhibitor.

Among those with available information, 60 of 75 individuals (80.0%) had elevated troponin levels, whereas 39 of 62 (62.9%) had at least 1 elevated biomarker of inflammation (C-reactive protein, erythrocyte sedimentation rate, or D-dimer). Electrocardiogram abnormalities were identified in 49 of 75 individuals (65.3%), with ST-segment and/or T-wave changes predominant in 40 adolescents (81.6%) and findings of dysrhythmia in 9 adolescents (18.4%). A total of 62 adolescents had a documented echocardiogram; the results from 12 scans (19.4%) were reported as abnormal, and 2 adolescents (3.2%) had a left ventricular ejection fraction (LVEF) of less than 50% at the time of the acute episode. Overall, 8 individuals (12.9%) were noted to have a pericardial effusion through diagnostic imaging. Seven cases of pericardial effusion (87.5%) were identified through echocardiogram, and 1 (12.5%) was identified on computed tomography.

Almost three-quarters of individuals (57 [74.0%]) were treated with over-the-counter NSAIDs only (Table 2). A further 8 adolescents (11.7%) were treated with NSAIDs and colchicine, and 11 (14.3%) received no treatment. None were treated with intravenous immunoglobulin or corticosteroids.

Among those with myocarditis or pericarditis after dose 1 (n = 26), 5 adolescents (19.2%) had a previous PCR-confirmed SARS-CoV-2 infection, with a median (IQR) time of 162 (86-259) days between infection and vaccination (Table 2). Of those with a reported event after dose 2, none had a previous confirmed SARS-CoV-2 infection. No individuals with a reported event after either dose had a previous history of myocarditis or pericarditis.

Overall, the reported incidence was significantly higher after dose 2 as compared with dose 1 (6.3; 95% CI, 4.7-8.3 vs 3.1; 95% CI, 2.0-4.5 per 100 000 doses administered, respectively) Table 3. The reported incidence also tended to be higher among male adolescents compared with female adolescents and among those aged 16 to 17 years (5.3; 95% CI, 3.0-8.8) compared with those aged 12 to 15 years (1.9; 95% CI, 1.0-3.5) (Table 3). The highest incidence was reported among male adolescents aged 16 to 17 years after dose 2 (15.7 per 100 000; 95% CI, 9.7-23.9). When we excluded the 5 individuals with a previous history of documented SARS-CoV-2 infection, the reported incidence after dose 1 decreased from 3.1 to 2.5 per 100 000 (95% CI, 1.5-3.8). When we restricted our analysis to events that occurred within 7 days of vaccine administration (65 of 77 total events), reported incidence decreased; however, the trends across dose number, sex, and age group remained similar to the overall findings (Table 3).

**Table 3.** Reported Incidence per 100 000 Doses Administered by Dose Number, Age, and Sex, Overall and Events Within 7 Days of Vaccination

Sex	Age, y	Dose 1			Dose 2		
		Events	Doses	Incidence per 100 000 (95% CI)	Events	Doses	Incidence per 100 000 (95% CI)
**Overall**							
Female	12-15	3	275 762	1.1 (0.2-3.2)	5	262 886	1.9 (0.6-4.4)
	16-17	4	139 279	2.9 (0.8-7.4)	2	132 523	1.5 (0.2-5.5)
	Total	7	415 041	1.7 (0.7-3.5)	7	395 409	1.8 (0.7-3.6)
Male	12-15	8	288 817	2.8 (1.2-5.5)	23	275 151	8.4 (5.3-12.5)
	16-17	11	142 155	7.7 (3.9-13.8)	21	134 065	15.7 (9.7-23.9)
	Total	19	430 972	4.4 (2.7-6.9)	44	409 216	10.8 (7.8-14.4)
Total	12-15	11	565 670	1.9 (1.0-3.5)	28	539 071	5.2 (3.5-7.5)
	16-17	15	282 054	5.3 (3.0-8.8)	23	267 178	8.6 (5.5-12.9)
Total		26^a^	847 724	3.1 (2.0-4.5)^b^	51	806 249	6.3 (4.7-8.3)
**Events within 7 d of vaccination**							
Female	12-15	1	275 762	0.4 (0-2.0)	5	262 886	1.9 (0.6-4.4)
	16-17	2	139 279	1.4 (0.2-5.2)	2	132 523	1.5 (0.2-5.5)
	Total	3	415 041	0.7 (0.1-2.1)	7	395 409	1.8 (0.7-3.6)
Male	12-15	6	288 817	2.1 (0.8-4.5)	21	275 151	7.6 (4.7-11.7)
	16-17	7	142 155	4.9 (2.0-10.1)	21	134 065	15.7 (9.7-23.9)
	Total	13	430 972	3.0 (1.6-5.2)	42	409 216	10.3 (7.4-13.9)
Total	12-15	7	565 670	1.2 (0.5-2.5)	26	539 071	4.8 (3.2-7.1)
	16-17	9	282 054	3.2 (1.5-6.1)	23	267 178	8.6 (5.5-12.9)
Total		16	847 724	1.9 (1.1-3.1)	49	806 249	6.1 (4.5-8.0)

^a^ Includes 1 male adolescent aged 12 to 15 years, 1 female adolescent aged 16 to 17 years, and 3 male adolescents aged 16 to 17 years who had an event following dose 1 who had a history of reverse transcription–polymerase chain reaction–confirmed SARS-CoV-2 infection; of these, 3 had symptom onset within 7 days after vaccine dose.

^b^ When the 5 individuals with previous SARS-CoV-2 infection were removed, the incidence was 2.5 (95% CI 1.5-3.8) per 100 000.

For the 51 reports after dose 2, the incidence was similar by interdose interval among individuals aged 12 to 15 years (Figure). However, among those aged 16 to 17 years, the reported incidence was significantly higher in those who received dose 2 within 30 days of dose 1 (21.3 per 100 000; 95% CI, 11.0-37.2) as compared with those with an interval of 31 to 55 days and appeared higher when compared with 56 days or more, although CIs overlapped (Figure). This reported incidence was primarily driven by male adolescents, who accounted for 91.3% (21 of 23) of all events after dose 2 in those aged 16 to 17 years.

**Figure.**
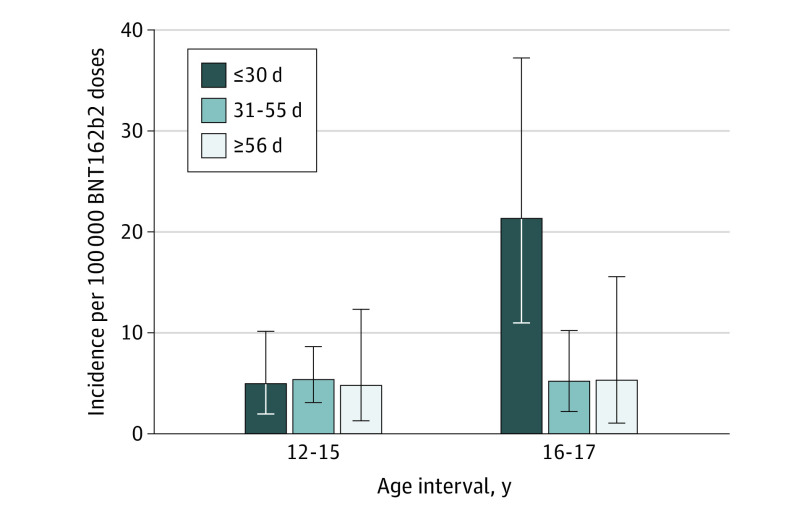
Reported Incidence After Dose 2 per 100 000 Doses Administered by Age Group and Interdose Interval

Finally, we found a statistically significantly higher rate of observed events compared with expected events for male adolescents aged 12 to 15 years and 16 to 17 years after dose 1; all rate differences reported are per 100 000 doses (age 12-15 years: rate difference, 2.0; 0.3-3.7; age 16-17 years: rate difference, 4.7; 95% CI, 0.9-8.4) and dose 2 (age 12-15 years: rate difference, 7.6; 95% CI, 4.3-10.8; age 16-17 years, rate difference, 15.4; 95% CI, 8.6-22.2), as well as female adolescents aged 12 to 15 years after dose 2 (rate difference, 1.9; 95% CI, 0.2-3.6). However, there were some strata with a very small number of events (Table 4). The findings were unchanged when the analysis was repeated after removing 2 reported events that had not been seen in the emergency department or hospitalized (eTable in Supplement 1).

**Table 4.** Rate Difference Comparing Observed to Expected Events of Myocarditis or Pericarditis Using a 7-Day Risk Window After Dose 1 or 2 of BNT162b2 COVID-19 Messenger RNA Vaccine by Age Group, Sex, and Dose (n = 65)

Sex	Age group, y	Rate difference (95% CI) per 100 000 doses	
		Dose 1	Dose 2
Female	12-15	0.3 (−0.4 to 1.1)	1.9 (0.2 to 3.6)
	16-17	1.4 (−0.6 to 3.4)	1.5 (−0.7 to 3.6)
Male	12-15	2.0 (0.3 to 3.7)	7.6 (4.3 to 10.8)
	16-17	4.7 (0.9-8.4)	15.4 (8.6-22.2)

## Discussion

In this cohort study of passive vaccine safety surveillance data from Ontario, Canada’s most populous province, results suggest that the reported incidence of myocarditis or pericarditis after BNT162b2 vaccine was very rare (ie, <0.01% based on standard pharmacogivilance definitions)^28^ in adolescents overall. Although incidence of myocarditis or pericarditis was higher in those aged 16 to 17 years than in those aged 12 to 15 years, this outcome was still rare (ie, <0.1%).^28^ Almost all episodes were seen in an emergency department, fewer than one-half of adolescents were admitted to hospital, and those who were hospitalized experienced a short length of stay (median duration: 1 day).

The clinical presentations of cardiac-specific symptoms in this cohort were consistent with other reports, with chest pain being the most frequently reported symptom on presentation. Fever was reported as a nonspecific symptom in 18% of individuals, slightly lower than proportions reported in similar studies evaluating populations younger than 21 years.^14,16,29,30^ Although troponin levels were elevated in almost all adolescents diagnosed with myocarditis or myopericarditis, further analyses examining troponin levels (ie, median value and range) were not feasible due to insufficient details regarding the troponin assay (ie, troponin I or T assay as well as laboratory-specific reference ranges). For individuals who had an echocardiogram completed, similar to other reports in adolescents, a large proportion of the current cohort had normal results, indicating no ventricular dysfunction or regional wall motion abnormalities.^14,31^ Cardiac magnetic resonance imaging studies were not routinely ordered as part of the cardiac evaluation in the passive reports included in this analysis. Overall, many of the cases of myocarditis or pericarditis were mild and required either no treatment or were managed conservatively with NSAIDs, similar to what has been reported in other studies.^14,15,31,32^ However, these studies reflect data from the acute episode, and less is known about the long-term outcomes of individuals with myocarditis or pericarditis after COVID-19 vaccination, although data are now emerging.^33^

Our findings of the reported incidence of myocarditis or pericarditis after vaccination, including the difference by more granular age groups among adolescents, are in line with data from other jurisdictions that have primarily used BNT162b2 for their adolescent program. In Israel, where a 21-day interval between dose 1 and 2 has been used, the incidence of myocarditis only among male adolescents aged 16 to 19 years was 15 per 100 000 after dose 2 as compared with 6.2 per 100 000 in male adolescents aged 12 to 15 years, as of January 17, 2022.^13^ In the US, where a 21-day interval between doses was also used, incidence data from the Vaccine Adverse Event Reporting System also demonstrated higher risk for individuals aged 16 to 17 years than for those aged 12 to 15 years for both male and female adolescents.^1^ The rate of myocarditis within a 7-day risk interval in this study was 11 per 100 000 after dose 2 for those aged 16 to 17 years compared with 7.1 in those aged 12 to 15 years. Similar to the current study, the observed cases in this US analysis exceeded the expected number of events for both sexes and both adolescent age groups after dose 2 (and for male adolescents after dose 1). However, as the historical incidence data used to compare observed vs expected events will differ across data sources and jurisdictions, expected case rates and rate differences will vary.^1,34,35,36^ Given the rarity of myocarditis or pericarditis outcomes, the absolute difference comparing observed to expected cases was low; in our study, the incidence of excess number of cases was 2 or less per 100 000 for female adolescents (both age strata and after both vaccine doses) and male adolescents aged 12 to 15 years after dose 1. The incidence of excess cases was higher for male adolescents aged 12 to 15 years after dose 2 (7.6 per 100 000), male adolescents aged 16 to 17 years (4.7 per 100 000), and for male adolescents aged 16 to 17 years after dose 2 (15.4 per 100 000).

The highest rates of myocarditis or pericarditis after vaccination and the largest excess rates were observed in male adolescents aged 16 to 17 years in the current study. This group also had the highest background incidence of myocarditis or pericarditis, where variations by age and sex have been observed; incidence was higher in individuals aged 16 to 17 years than in individuals aged 12 to 15 years and higher in male adolescents than in female adolescents. Although the reasons for male predominance of myocarditis or pericarditis is unknown, hormonal differences (ie, testosterone) are postulated to play a role.^36^ Our findings also suggest that a longer interdose interval may reduce the risk of myocarditis or pericarditis in individuals aged 16 to 17 years.^25^ We also observed that of the reported myocarditis or pericarditis events in our sample that occurred among individuals with a history of laboratory-confirmed COVID-19 infection, all occurred after dose 1; this finding merits further exploration in future studies and may have implications for recommended timing of first doses after infection.

### Strengths and Limitations

This study has several strengths. First, we have access to data on all COVID-19 vaccines as well as all AEFI reports for the province, which is strengthened by Ontario’s high 2-dose coverage in individuals aged 12 to 17 years (73% in November 2021). This allowed us to not only examine rates by narrower age ranges but also to summarize the clinical features of myocarditis or pericarditis events collected as part of enhanced passive surveillance in Ontario that was established in June 2021. Additionally, all reports and associated medical records collected as part of the AEFI investigation were reviewed by specialized nurses and physicians, which allowed us to include only those meeting BC case definitions 1 through 3. Further, Ontario has differed in the rollout of vaccine compared with other jurisdictions, specifically in the variation in timing between dose 1 and 2. This provided us a unique opportunity to estimate the reported incidence of myocarditis or pericarditis by interdose intervals, which may not be possible in most other jurisdictions and may have implications for program decision-making; this includes balancing the benefits of a longer interdose interval against the epidemiologic risk of infection prior to receiving dose 2. 

Limitations of this analysis include small numbers of events in some groups, leading to wide CIs. Additionally, public awareness of this safety signal, along with the enhanced surveillance, may have stimulated reporting of myocarditis or pericarditis events to the passive surveillance system. However, our age-specific reported incidence rates are in line with other published estimates for adolescents.^1,13,14,32^ Any stimulated reporting is also very unlikely to play a role in our findings of interdose interval, as only 0.2% of individuals aged 12 to 17 years had received dose 2 by June 1, 2021 (the start of enhanced surveillance). Additionally, if individuals (asymptomatic or symptomatic of COVID-19 infection) did not seek testing, we would not be able to account for these infections in our analyses. Finally, our study was limited to events of myocarditis or pericarditis reported to Ontario’s passive vaccine safety surveillance system, but the specific etiology is not possible to establish from passive surveillance. Further, the AEFI system does not include clinical reports of myocarditis or pericarditis associated with other causes, including infection with SARS-CoV-2, or other outcomes, precluding the ability to contrast this vaccine safety assessment with the numerous benefits of vaccination in adolescents including the prevention of infection-associated hospitalization,^37,38^ myocarditis^8,39^ and multisystem inflammatory syndrome in children,^40^ and potentially post-COVID conditions (ie, long COVID).^41,42,43^ The benefit of vaccination in preventing complications of infection has been demonstrated to outweigh the risk of myocarditis across age groups.^36,44,45,46^

## Conclusions

Results of this cohort study suggest that there was variation in the reported incidence of myocarditis or pericarditis after BNT162b2 vaccine among adolescent age groups, with higher incidence observed in individuals aged 16 to 17 years than in those aged 12 to 15 years. However, the risk of these events after vaccination remains very rare and needs to be considered in the context of the reduced risk of COVID-19 infection and associated outcomes as a result of protection from vaccination. Additional long-term follow-up on individuals with myocarditis or pericarditis after vaccination is needed.

## Notes

Supplement 1. **eTable.** Rate Difference Comparing Observed Events to Expected Events of Myocarditis/Pericarditis Using a 7-Day Risk Window Following Dose 1 or 2 of BNT162b2 COVID-19 mRNA Vaccine by Age Group, Sex, and Dose, Restricted to Observed Events Known to Have Been Seen in the Emergency Department and/or Have Been Hospitalized (n = 63)

Supplement 2. Data Sharing Statement
