# Supplementary material for: Myocarditis or Pericarditis Events After BNT162b2 Vaccination in Individuals Aged 12 to 17 Years in Ontario, Canada
Source: JAMA Pediatr. 2023 Feb 27;177(4):410–8. doi: 10.1001/jamapediatrics.2022.6166 (PMC9972235; doi:10.1001/jamapediatrics.2022.6166)
Supplement: Supplement 1. — eTable. Rate Difference Comparing Observed Events to Expected Events of Myocarditis/Pericarditis Using a 7-Day Risk Window Following Dose 1 or 2 of BNT162b2 COVID-19 mRNA Vaccine by Age Group, Sex, and Dose, Restricted to Observed Events Known to Have Been Seen in the Emergency Department and/or Have Been Hospitalized (n = 63) [file jamapediatr-e226166-s001.pdf]

Supplementary Online Content

Buchan SA, Alley S, Seo CY, et al. Myocarditis or pericarditis events after BNT162b2 vaccination in individuals aged 12 to 17 years in Ontario, Canada. *JAMA Pediatr*. Published online February 27, 2023. doi:10.1001/jamapediatrics.2022.6166

eTable. Rate Difference Comparing Observed Events to Expected Events of Myocarditis/Pericarditis Using a 7-Day Risk Window Following Dose 1 or 2 of BNT162b2 COVID-19 mRNA Vaccine by Age Group, Sex, and Dose, Restricted to Observed Events Known to Have Been Seen in the Emergency Department and/or Have Been Hospitalized (n = 63)

This supplementary material has been provided by the authors to give readers additional information about their work.

© 2023 Buchan SA et al. *JAMA Pediatrics.*

eTable. Rate Difference Comparing Observed Events to Expected Events of Myocarditis/Pericarditis Using a 7-Day Risk Window Following Dose 1 or 2 of BNT162b2 COVID-19 mRNA Vaccine by Age Group, Sex, and Dose, Restricted to Observed Events Known to Have Been Seen in the Emergency Department and/or Have Been Hospitalized (n = 63)

Sex	Age group (years)	Rate difference (95% CI) per 100 000 doses	
		Dose 1	Dose 2
Female	12-15	0.3 (-0.4 – 1.1)	1.9 (0.2 – 3.6)
	16-17	1.4 (-0.6 – 3.4)	1.5 (-0.7 – 3.6)
Male	12-15	1.7 (0.1 – 3.2)	7.6 (4.3 – 10.8)
	16-17	3.9 (0.5 – 7.4)	15.4 (8.6 – 22.2)

© 2023 Buchan SA et al. *JAMA Pediatrics.*
