# Supplementary material for: Myocarditis or Pericarditis Events After BNT162b2 Vaccination in Individuals Aged 12 to 17 Years in Ontario, Canada
Source: JAMA Pediatr. 2023 Feb 27;177(4):410–8. doi: 10.1001/jamapediatrics.2022.6166 (PMC9972235; doi:10.1001/jamapediatrics.2022.6166)
Supplement: Supplement 2. — Data Sharing Statement [file jamapediatr-e226166-s002.pdf]

Buchan SA, Alley S, Seo CY, et al. Myocarditis or pericarditis events after BNT162b2 vaccination in individuals aged 12 to 17 years in Ontario, Canada. *JAMA Pediatr.* Published online February 27, 2023. doi:10.1001/jamapediatrics.2022.6166

Data Sharing Statement

Data
Data available: No
